# Cost Index Model for the Process Performance Optimization of Micro-EDM Drilling on Tungsten Carbide

**DOI:** 10.3390/mi8080251

**Published:** 2017-08-17

**Authors:** Gianluca D’Urso, Claudio Giardini, Mariangela Quarto, Giancarlo Maccarini

**Affiliations:** Department of Management, Information and Production Engineering (DIGIP), University of Bergamo, 24044 Dalmine (BG), Italy; gianluca.d-urso@unibg.it (G.D.); mariangela.quarto@unibg.it (M.Q.); giancarlo.maccarini@unibg.it (G.M.)

**Keywords:** micro-electrical discharge machining (micro-EDM), tungsten carbide, micro-drilling, process performance, model

## Abstract

The present work deals with the execution of through micro-holes on tungsten carbide plates using a micro-electrical discharge machining (micro-EDM) machine. The experiments were carried out by varying peak current, voltage and frequency in order to achieve suitable technology windows. Tubular electrodes, made of two different materials (tungsten carbide and brass), were used. The investigation focuses on the influence of variable process parameters on the process performances and their optimization. The performance indicators taken into account were Material Removal Rate (MRR) and Tool Wear Ratio (TWR). A general model based on a cost index was defined for the process performances optimization and the optimal conditions were identified through the minimization of the objective function.

## 1. Introduction

The current trends in manufacturing technology often deal with the miniaturization of products and components and, consequently, the need for products containing micro-features has shown a noticeable and continuous growth in many fields of application. At the same time, materials with distinctive physical and mechanical properties, such as tungsten carbide and its composites, titanium based alloys, nickel based alloys, tool steels and other super alloys, have been developed to meet the needs of extreme applications. In general, these materials have high performing properties in terms of hardness, toughness, low heat sensitivity, high fatigue and corrosion resistance with respect to other more common materials, even though they are often more difficult to be machined. Among the difficult-to-cut materials, tungsten carbide (WC) is an extremely hard material widely used in manufacturing because of its superior wear and corrosion resistance. Nowadays, WC and its composite (WC–Co) are of great interest in the production of cutting tools, dies and other special tools and components [1]. Therefore, significant research has been carried out for machining this material using both conventional and non-conventional machining processes. Even if it is possible to machine this material with some conventional methods, the high accuracy required in machining complex shapes cannot be achieved. Attempts have been made to machine this material with turning, indicating limited success when employing boron nitride tools with a cutting speed of 20–30 m/min, a feed rate of 0.2–0.25 mm/rev and a cutting depth of 0.1–0.5 mm [2]. Moreover, high-speed milling was found to be difficult to apply in machining cemented carbide due to the material’s extreme hardness and strength. In addition, finishing operations might become necessary to remove cracks and to produce a good surface quality when this machining method is used [2,3].

In contrast, non-conventional technologies have proven to be particularly suitable, not only to overcome the problems related to very hard materials, but also for applications where dimensional accuracy and complex geometries are primary requirements. Among such non-conventional methods, electro-discharge machining (EDM) is a technique widely used in industry for high-precision machining of all types of conductive materials such as: metals, metallic alloys, graphite and even some ceramic materials, of whatsoever hardness. In particular, EDM is one of the most performing methods capable of machining WC–Co composites [2,3,4]. Moreover, it is considered one of the most important technologies in micro-drilling because of its effectiveness in achieving very small and very high aspect ratio burr-free micro-holes. The need for products containing micro-holes has shown a remarkable growth [5,6], since they are widely used for the production of several industrial components such as medical and optical devices, turbine blades cooling channels and diesel fuel injection nozzles.

Complex electrothermal phenomena, surface irregularities, interaction between successive discharges and the presence of debris make the micro-EDM process complex; for this reason, a complete and accurate physical modelling of the process is very difficult to be developed. To achieve the best machining performances and to increase the production while reducing the machining time, the correct selection of the process parameters is required. Usually, the process parameters are fixed based on experience or on handbook values, but this approach does not ensure that the chosen process parameters accomplish optimal or near-optimal machining performances. In some cases, the Taguchi method and similar techniques have been applied to carry out parametric design, even though they are limited to the optimization of only one response at a time; as such, in case of multiple responses these methods do not work [7,8]. Therefore, a multiple response optimization method is necessary to obtain the best parametric combination for the micro-EDM process [9]. 

The most used models are based on natural rules. For example, in the Genetic Algorithm (GA), the genetic laws are turned into a mathematical model used to optimize the tool, while in the Artificial Neural Network (ANN), the study of neurons and their functionality is involved. In the same way, in the Ant Colony Optimization (ACO) and in the Artificial Bee Colony (ABC) algorithms, the social behavior of ants and bees are imitated. Similarly, the Biogeography-Based Optimization (BBO) algorithm considers the mathematics of biological distribution of different species to work out complex optimization problems [10]. 

Somashekhar et al. [11] used an ANN algorithm for analyzing the material removal rate to establish the parameter optimization model and, at the same time, they used the GA algorithm to determine the optimal process parameters. Panda [12] simulated an ANN to characterized surface roughness, material removal rate and microhardness values of machined surface with respect to current and pulse duration. Assarzadeh et al. [13] presented an integrated network-based approach for the prediction and optimal selection of machining parameters in die-sinking EDM. Peak current, pulse on time and voltage were selected as the network inputs, while material removal rate and surface roughness were the output parameters of the model. Chiang [14] developed a response surface method based on mathematical models to investigate the influences of discharge current, pulse on time, duty factor and voltage on material removal rate, tool wear ratio and surface roughness in the EDM. In the same way, Çaydaş et al. [15] considered pulse on time and peak current as the most significant parameters of the die-sinking EDM process and developed a model for electrode wear. Mukherjee et al. [16] applied a BBO algorithm to determine the optimal combination of several machining parameters for improved EDM process performances. They took into account the mathematical models of various responses, such as material removal rate, tool wear ratio and surface roughness, seeking the optimal solution. When this algorithm is applied to optimize the responses, it calculates the optimal values of EDM process parameters that will carry out the best results. Comparative analyses to other optimization algorithms (GA, ACO, ABC) show that the BBO algorithm gives the best results.

Despite the great developments in EDM technology, there has been limited research activity concerning the economic aspects of the process. For example, in the literature, a model for predicting the manufacturing cost of micro-EDM process was developed in Reference [17] and a comparative analysis of different mathematical methods is made in Reference [18], but, in both these works, there is not a correlation between the optimization of the process performances and the production cost.

The present paper aims to evaluate the influence of variable process parameters on process performance for the production of micro-holes on tungsten carbide plates. Peak current, open circuit voltage and discharges frequency are varied to achieve low and high limits to identify the technology window suitable for the specific combination between workpiece and electrode materials. Moreover, a cost index model was proposed for the optimization of the process performance.

## 2. Experimental Procedure

Experimental tests based on the execution of through micro-holes were carried out using a micro-EDM machine Sarix SX-200 (Sarix SA, Sant’Antonino, Switzerland). Tungsten carbide plates with thickness equal to 3 mm were used as workpiece materials. The experimental campaign was carried out by varying several process parameters, namely peak current (*I*), voltage (*V*), and frequency (*F*). Tubular electrodes made of tungsten carbide and brass, with an outer diameter equal to 0.3 mm and an inner diameter equal to 0.12 mm, were used. Hydrocarbon oil was used as a dielectric and an internal electrode washing pressure equal to 30 bar was set for all the experiments. 

For each tool material type, the combination of the process parameters and the experimental sequence were defined based on a Central Composite Design—CCD (Factors: 3, Base runs: 20, Base blocks: 1, Replicates: 5, Total runs: 100, Total blocks: 1). 

Based on this approach, 15 combinations of process parameters were defined (Table 1). 

Preliminary experimental tests were performed for the identification of a suitable technology window, in terms of minimum and maximum values of peak current, voltage, and frequency, for each combination of workpiece–electrode materials.

The fixed parameters and the ranges of the variable parameters differ for the two electrode materials; this solution was used for testing suitable technology windows (in other words, ranges of suitable process parameters) for both materials. The execution order of the experiments was randomized to avoid possible systematic errors. The fixed process parameters used for the tests are reported in Table 2. At the end of each hole drilling operation, the electrode tip was cut using the wire EDM unit to restore a standard initial condition.

It is important to remark that in the Sarix EDM machine, some process parameters are expressed as indexes. In particular, the instantaneous values cannot be set, because the machine presents an autoregulating system. In particular, peak current is an index defining the maximum current that the pulse can reach. This index must be set up respecting the required roughness for the part to be machined and to avoid the burns and coarse melting that would be inevitable with pulses of uncontrolled current. The peak current parameter regulates the maximum amplitude of the long pulses and of the high-power pulses. A similar consideration can be made for gap; in this case, the value is proportional to the distance between the electrode and the workpiece. The higher the gap index, the higher then actual distance between the two elements. Furthermore, the regulation index represents a certain regulation management algorithm for the setting of the electrode movement. Finally, the energy level is an index that establishes the shape of the pulse.

## 3. Evaluation of Process Performance

### 3.1. Performance Criteria

*MRR* and *TWR* were selected as performance criteria for the micro-holes drilling. Due to the debris movement and secondary discharges occurring between the hole wall and the electrode side, the machined hole is not perfectly cylindrical; therefore, the diameter was measured at both the top (*D_top_*) and the bottom (*D_bottom_*) of each hole through an optical measuring microscope with a magnification of 100×.

**Figure 1:**
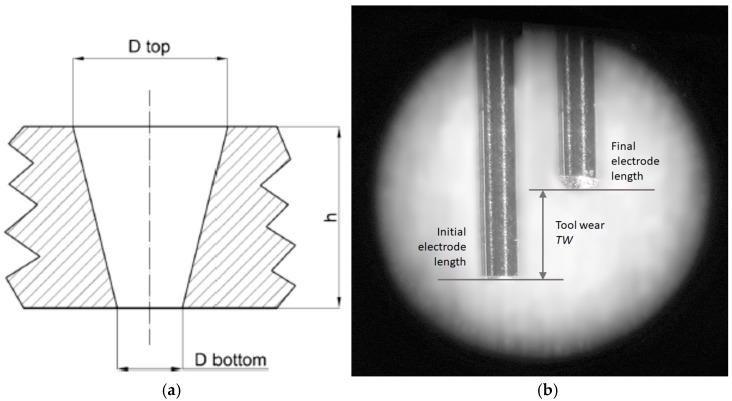
Hole geometry scheme (**a**) and electrode tool wear (TW) definition (**b**).

*MRR* (mm^3^/s) was calculated as the rate of material removed from the workpiece (*MRW* (mm^3^), estimated as the frustum volume, as showed in Figure 1a, with respect to the machining time *t* (s) recorded by the EDM system (Equation (1)).

(1) $$MRR=\frac{MRW}{t}=\frac{\pi h\left(D_{top}^{2}+D_{top}\cdot D_{bottom}+D_{bottom}^{2}\right)}{12t}$$

where *h* is the thickness of the plate.

*TWR* (Equation (2)) was calculated as the ratio between the material removed from the electrode (*MRT*, Equation (3)) and the material removed from the workpiece. The material removed from the electrode tool is measured through a touching procedure executed in a reference position: the length of the electrode is taken before and after the single drilling operation (Figure 1b).

(2) $$TWR=\frac{MRT}{MRW}$$

(3) $$MRT=\frac{\pi TW\left(D_{ext}^{2}-D_{int}^{2}\right)}{4}$$

where *D_ext_* is the external diameter and *D_int_* is the internal electrode diameter of the tubular electrode; *TW* is equal to the length of electrode worn for each hole.

### 3.2. Cost Index

In general, the optimal machining performances are expected to be characterized by high *MRR* and low *TWR*. Since these two indicators have opposite behaviors, they are combined in a single index starting from the machining cost *C* (€):

(4) $$C=C_{0}\cdot t+C_{t}\cdot MRT$$

where $C_{0}$ (€/s) is the cost per time unit of the machining operation, *t* (s) is the machining duration, $C_{t}$ (€/mm^3^) is the tool cost related to the material removed from the electrode (i.e., the *MRT* (mm^3^)). This index can be expressed as function of *MRR* and *TWR* using Equation (1) and Equation (2) as reported below:

(5) $$t=\frac{MRW}{MRR}$$

(6) MRT=MRW·TWR

Then, the cost index per unitary volume of removed material (*CI*) can be obtained by replacing Equations (5) and (6) in Equation (4):

(7) $$CI=\frac{C}{MRW\cdot C_{0}}=\frac{1}{MRR}+\frac{C_{t}}{C_{0}}\cdot TWR$$

Equation (7) represents the objective function to be minimized. To describe *MRR* and *TWR* data coming from the experiments, the regression equations, as function of variable process parameters (*I*, *V*, and *F*), were used. It is important to remark that these regression equations are different for each combination of workpiece-electrode materials. The minimization of *CI*, which must be carried out under the physical constraints of *I*, *V* and *F* values, can be expressed as follows:

(8) $$Min\;CI\left(\begin{array}{c} Under\\ constraints \end{array}\right)\{\begin{array}{c} MRR\left(I,\;V,\;F\right)\\ TWR\left(I,\;V,\;F\right)\\ I_{min}\leq I\leq I_{max}\\ V_{min}\leq V\leq V_{max}\\ F_{min}\leq F\leq F_{max} \end{array}$$

To minimize the cost index (CI), it is necessary to identify the critical points of the function. In particular, the first partial derivatives $\left(\frac{\partial CI}{\partial I};\frac{\partial CI}{\partial V};\frac{\partial CI}{\partial F}\right)$ were calculated and the critical points were obtained by simultaneously satisfying the equations:

(9) $$Min\left(CI\right)=\{\begin{array}{c} \frac{\partial CI}{\partial I}\left(I,V,F\right)=0\\ \frac{\partial CI}{\partial V}\left(I,V,F\right)=0\\ \frac{\partial CI}{\partial F}\left(I,V,F\right)=0 \end{array}$$

Since the critical points could be either minimum, maximum or saddle point, the second order partial derivatives ($\frac{\partial^{2}CI}{\partial I^{2}};\frac{\partial^{2}CI}{\partial V^{2}};\frac{\partial^{2}CI}{\partial F^{2}};\frac{\partial^{2}CI}{\partial I\partial V};\frac{\partial^{2}CI}{\partial I\partial F};\frac{\partial^{2}CI}{\partial V\partial I};\frac{\partial^{2}CI}{\partial V\partial F};\frac{\partial^{2}CI}{\partial F\partial I};\frac{\partial^{2}CI}{\partial F\partial V})$ were calculated and the 3 × 3 Hessian matrix, representing the local curvature of the function in several variables, was built. The Hessian matrix is a square matrix of second-order partial derivatives of a scalar-valued function. It describes the local curvature of a function of several variables.

(10) $$H\left(I,V,F\right)=\left[\begin{array}{ccc} \frac{\partial^{2}CI}{\partial I^{2}} & \frac{\partial^{2}CI}{\partial I\partial V} & \frac{\partial^{2}CI}{\partial I\partial F}\\ \frac{\partial^{2}CI}{\partial V\partial I} & \frac{\partial^{2}CI}{\partial V^{2}} & \frac{\partial^{2}CI}{\partial V\partial F}\\ \frac{\partial^{2}CI}{\partial F\partial I} & \frac{\partial^{2}CI}{\partial F\partial V} & \frac{\partial^{2}CI}{\partial F^{2}} \end{array}\right]$$

The nature of critical points was defined by calculating the matrix determinant for these points; if the Hessian is positive definite and $\frac{\partial^{2}CI}{\partial I^{2}}>0$ at critical point, the function will attain an isolated minimum. In this case, the critical point represents the combination of process parameters *I*, *V,* and *F*, minimizing the cost index *CI*, that is the most performant material removal rate (*MRR*) and tool wear ratio (*TWR*) combination.

## 4. Analysis of the Results

**Figure 2:**
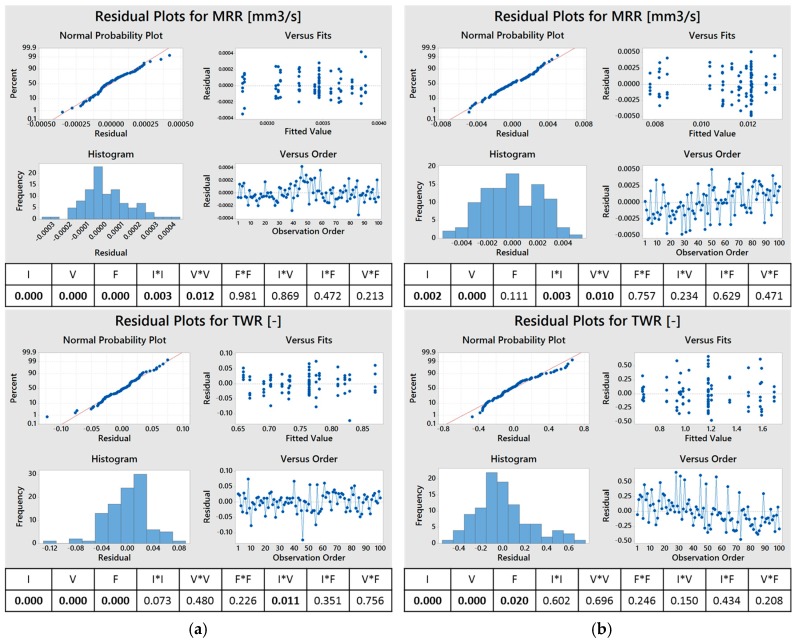
Residual plots for material removal rate (*MRR*) and tool wear ratio (*TWR*) obtained using tungsten carbide (**a**) and brass (**b**) electrodes. The *p*-values are reported in the tables below the graphs.

The response surface analysis was applied to the experimental results. Figure 2 reports the *p*-values derived from analysis of variance (ANOVA), calculated using Minitab software, for *MRR* and *TWR* for both the combinations of workpiece-electrode materials. The parameters are considered to influence the process when the *p*-value is less than 2.5%, since a confidence interval of 97.5% was used.

As a general remark, all the performance indexes resulted to be influenced by peak current and voltage. In some cases, the second order parameters and some interactions also showed a negligible effect. The residuals, in all cases, were demonstrated to be normally distributed and randomly scattered with an average value near to zero. Figure 2 shows the residual plots of *MRR* and *TWR* obtained using a tungsten carbide electrode (a) and a brass electrode (b).

**Figure 3:**
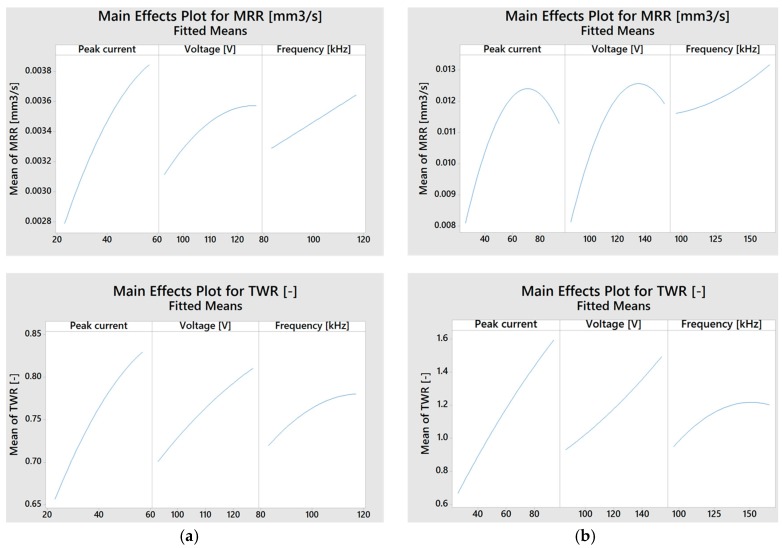
Main effects plots for material removal rate (*MRR*) and tool wear ratio (*TWR*) for tungsten carbide (**a**) and brass (**b**) electrodes.

Figure 3 shows the main effects plots (of both the electrode materials) for *MRR* and *TWR* as a function of the process parameters; in some cases, a relative maximum can be observed within the defined domain, while in other cases the curves show a progressive monotonous trend in the defined domain. The plot shows that the brass electrode always allows the machining to be obtained quickly compared to the tungsten carbide electrode, due to the higher electrical characteristics and the discharge energy transferred during the machining. From the point of view of the *TWR*, the brass electrode suffers higher wear and its variability range is wider with respect to the tungsten carbide electrode. These aspects are probably correlated to the high electrical conductivity and low melting point of the brass electrode.

**Figure 4:**
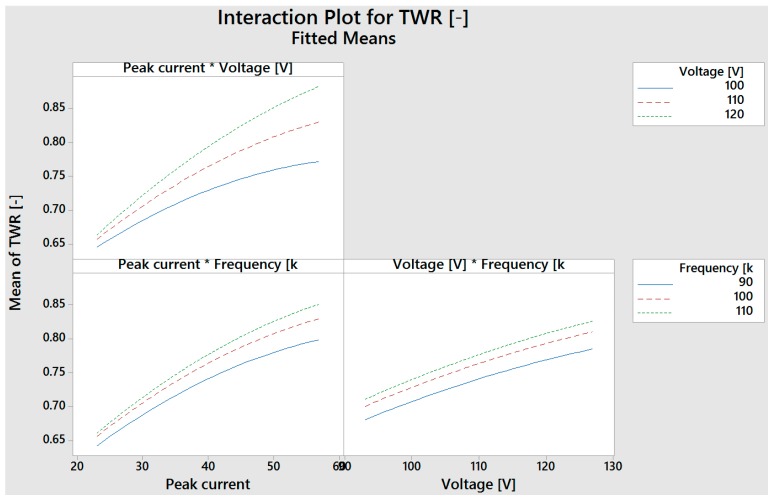
Interaction plots for tool wear ratio (*TWR*) obtained by the tungsten carbide electrode.

The interaction plots reported in Figure 4 show that the *TWR* obtained by using a tungsten carbide electrode is influenced by the interaction between the peak current (*I*) and the voltage (*V*); this is confirmed by the *p*-values. The other interaction plots show, according to the *p*-values, the non-influence of the interactions among the parameters on *MRR* and *TWR*.

## 5. Estimation of Cost Index (CI)

### 5.1. Tungsten Carbide Electrode

As previously mentioned, the cost index function to be minimized is (as reported in Equation (7)):

(11) $$CI=\frac{1}{MRR}+\frac{C_{t}}{C_{0}}\cdot TWR$$

The response surface analysis returned, as a result, the regression equation in coded units of *MRR* (Equation (12)) and *TWR* (Equation (13)) as function of the process parameters in which the influencing factors and interactions are considered:

(12) $$MRR=0.003466+0.000310I+0.000134V+0.000105F-0.000051I^{2}-0.000042V^{2}$$

(13) TWR=0.76367+0.05088I+0.03221V+0.01782F+0.01369VI

In this specific case, the estimated tool cost is about 1.90 €/mm^3^, while it was assumed that the cost per hour of the machine is equal to 70 €/h, corresponding to *C*_0_ equal to 0.019 €/s. *C*_0_ is the average cost to which some surveyed companies sell their EDM work time.

Moreover, the *CI* equation must be minimized according to the following constraints:

(14) 23≤I≤5793 V≤V≤127 V83 kHz≤F≤117 kHz

The critical points are identified, solving the system of equations given by the first order partial derivatives of *CI* equal to zero. The nature of the critical points is defined by the estimation of the Hessian matrix determinant.

By carrying out all the calculations, it is possible to find that the point in coded units (*I* = 1.7; *V* = 0.496; *F* = 1.7) is an absolute minimum for the *CI* function. This means that the cost index is minimized for the process parameters equal to:

*I* = 57, *V* = 115 V, *F* = 117 kHz.

**Figure 5:**
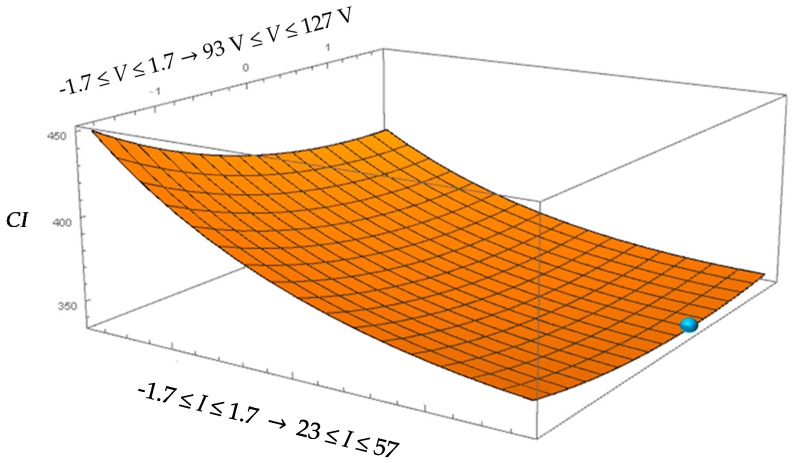
Surface plot of cost index (*CI*) for machining performed by the tungsten carbide electrode.

Figure 5 shows the surface generated corresponding to *CI* as function of *I* and *V* while considering the optimal value for *F* = 117 kHz. It is possible to observe that the optimal conditions are obtained in correspondence to the limits of the technological window (maximum values of process parameters suitable for this application).

### 5.2. Brass Electrode

For the machining performed by the brass electrode, the regression equations in coded units are obviously different with respect to the tungsten carbide electrode, specifically:

(15) $$MRR=0.12144+0.000934I+0.001111V-0.000849I^{2}-0.0007352V^{2}$$

(16) TWR=1.1774+0.2727I+0.1653V+0.0745F

In this case, the estimated tool cost is about 0.41 €/mm^3^ while C_0_ remains equal to 0.019 €/s and the constraints are expressed as follows:

(17) 26≤I≤9486 V≤V≤154 V96 kHz≤F≤160 kHz

For the brass electrode, the system of first partial derivatives and the estimation of the Hessian matrix determinant resulted in a minimum in the following point: *I* = 0.011; *V* = 0.378; *F* = −1.7 in coded units. This means that the cost index is minimized for the process parameters equal to:

*I* = 60.22; *V* = 127.56 V; *F* = 96 kHz.

**Figure 6:**
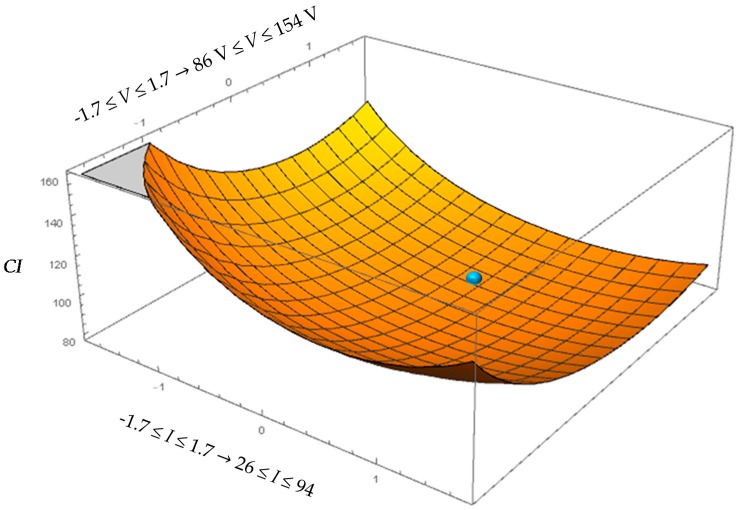
Surface plot of cost index (*CI*) for machining performed by the brass electrode.

Figure 6 represents the *CI* behaviour as function of *I* and *V* while considering the optimal value of *F* = 96 kHz.

## 6. Conclusions

In the present paper, the influence of *I*, *V*, *F* and of their interactions on the process performance in micro-EDM drilling was analyzed and presented in terms of *MRR* and *TWR*. The experiments were designed according to the CCD method. Since the optimal machining configuration is expected to be characterized by high *MRR* and low *TRW*, a cost index (*CI*), able to combine these two opposite effects, was defined. This index is based on both the tool cost and the cost per time unit of the machining operation where *MRR* and *TRW* have been expressed by means of the regression equations identified with the Response Surface Method. The minimization of the cost index allowed for the identification of the optimal working conditions.

The experiments showed that the ranges of the process parameters and their effects greatly depend on the materials combination. For this reason, the cost index equation also significantly differs and must be written for the specific couple of workpiece-electrode materials.

It is important to point out that the proposed method can be generalized and used successfully for both other combinations of workpiece-electrode materials and other EDM processes, such as milling and sinking.

## Figures and Tables

**Table 1:** Combination of process parameters for tungsten carbide (a) and brass (b) electrodes based on Central Composite Design (CCD).

(a)				(b)			
Tool Type	Tungsten Carbide			Tool Type	Brass		
	Peak Current (*I*)	Voltage (*V*)	Frequency (*F*)		Peak Current (*I*)	Voltage (*V*)	Frequency (*F*)
	(index)	(V)	(kHz)		(index)	(V)	(kHz)
WC1	40	110	83	BR1	26	120	130
WC2	30	100	90	BR2	40	100	110
WC3	50	100	90	BR3	40	140	110
WC4	30	120	90	BR4	40	100	150
WC5	50	120	90	BR5	40	140	150
WC6	40	93	100	BR6	60	120	96
WC7	23	110	100	BR7	60	86	130
WC8	40	110	100	BR8	60	120	130
WC9	57	110	100	BR9	60	154	130
WC10	40	127	100	BR10	60	120	160
WC11	30	100	110	BR11	80	100	110
WC12	50	100	110	BR12	80	140	110
WC13	30	120	110	BR13	80	100	150
WC14	50	120	110	BR14	80	140	150
WC15	40	110	117	BR15	94	120	130

**Table 2:** Fixed process parameters.

Electrode Material	Polarity	Ton (μs)	Gain	Gap (%)	Energy (Index)	Regulation
Tungsten Carbide	− (negative)	5	40	20	365	03-01
Brass	− (negative)	3.8	120	5	365	03-01
